# No Evidence for a Causal Link between Serum Uric Acid and Nonalcoholic Fatty Liver Disease from the Dongfeng-Tongji Cohort Study

**DOI:** 10.1155/2022/6687626

**Published:** 2022-03-15

**Authors:** Yuhan Tang, Yanyan Xu, Peiyi Liu, Cheng Liu, Rong Zhong, Xiao Yu, Lin Xiao, Min Du, Ling Yang, Jing Yuan, Youjie Wang, Weihong Chen, Sheng Wei, Yuan Liang, Xiaomin Zhang, Tangchun Wu, Meian He, Xiaoping Miao, Ping Yao

**Affiliations:** ^1^Department of Nutrition and Food Hygiene, Hubei Key Laboratory of Food Nutrition and Safety and the Ministry of Education (MOE) Key Lab of Environment and Health, School of Public Health, Huazhong University of Science and Technology, 13 Hangkong Rd, Wuhan, 430030 Hubei, China; ^2^Department of Epidemiology and Biostatistics and MOE Key Lab of Environment and Health, School of Public Health, Tongji Medical College, Huazhong University of Science and Technology, 13 Hangkong Rd, Wuhan, Hubei, China; ^3^Division of Gastroenterology, Department of Internal Medicine, Union Hospital, Tongji Medical College, Huazhong University of Science and Technology, Wuhan, Hubei, China; ^4^Institute of Occupational Medicine and MOE Key Lab of Environment and Health, School of Public Health, Tongji Medical College, Huazhong University of Science and Technology, 13 Hangkong Rd, Wuhan, 430030 Hubei, China

## Abstract

**Background and Aims:**

Elevated serum uric acid (SUA) is associated with an increased risk of nonalcoholic fatty liver disease (NAFLD); however, whether this association is causal is undetermined.

**Methods:**

Each participant from the Dongfeng-Tongji cohort study based on 27,009 retirees was interviewed face-to-face following a clinical examination. Covariance, logistic regression analysis, and instrumental variables were used to assess associations between SUA and (severity of) NAFLD and the causal link.

**Results:**

Among 8,429 subjects free of NAFLD at baseline, 2,007 participants developed NAFLD after 5 years of follow-up. The multivariable-adjusted odds ratio (OR) for NAFLD for individuals in the fourth quartile of SUA level versus those in the first was 1.71 (95% CI: 1.45-2.01, *P* for trend <0.001) and was more dramatic in women or normal-weight persons. Furthermore, SUA was materially associated with greater mean markers of hepatic necroinflammation and greater probabilities of fibrosis. In genetic analyses, both single nucleotide polymorphisms (rs11722228 to *SLC2A9* and rs2231142 to *ABCG2*) were pronouncedly associated with increased SUA concentrations, ranging from 0.19 to 0.22 mg/dl. No significant associations were observed between SNPs and potential confounders. No association was observed between the SUA-increasing allele and NAFLD, with an OR of 0.98 (95% CI: 0.90-1.08) per genetic score. This was not significantly different (*P* = 0.25) from what was expected (1.03, 95% CI: 1.03-1.03).

**Conclusions:**

SUA was positively associated with NAFLD incidence especially in female and normal-weight individuals and the suspected progression risk of newly developed NAFLD. However, the Mendelian randomization analyses lend no causal evidence, suggesting high SUA as a marker and not a cause of NAFLD.

## 1. Introduction

Nonalcoholic fatty liver disease (NAFLD), including even “benign” simple steatosis and steatohepatitis, poses a serious public health issue due to its high prevalence worldwide and poor long-term clinical outcomes [1]. Importantly, NAFLD can even stealthily progress to hepatocellular carcinoma after advanced fibrosis or cirrhosis with severe complications and high mortality. As speculated, NAFLD is one of the most common leading causes of hepatocellular carcinoma and is an indication for liver transplantation in the next decade [2].

Serum uric acid (SUA) has been reported to strongly reflect and even cause oxidative stress. Thus, the role of SUA in diseases has attracted increasing attention. The association of SUA and NAFLD has been confirmed in the last decade [3–6]. However, the prospective relationship between SUA and NAFLD has been less investigated. Although a positive association has been reported by two recent dose-response meta-analyses [7, 8] that pooled three prospective studies [3, 6, 9], limitations remain: Residual confounding cannot be ruled out. Furthermore, SUA has been linked with the incidence of cirrhosis-related hospitalization death of unknown etiology [10]; however, the significant association of SUA was confirmed in severe NAFLD (rather than mild or moderate NAFLD) and liver inflammatory alteration but not in fibrosis of NAFLD in other cross-sectional studies [5, 11].

Additionally, a recent study [12] proposed that NAFLD significantly increases the risk of incident hyperuricemia, indicating that the direction of causality could be reversed; namely, elevated SUA levels could be a consequence rather than a cause of NAFLD. Mendelian randomization analysis, which can overcome the influence of unmeasured confounding and reverse causation by using genotypes robustly associated with the risk factor of interest as instrumental variables, can be applied to test causality [13]. In the literature, the evidence of a causal association of SUA with NAFLD is lacking. Thus, using data from retirees from the Dongfeng-Tongji (DFTJ) cohort study with 5 years of follow-up, we aimed to meticulously examine the cause-and-effect association between SUA and (suspected progression of) NAFLD.

## 2. Methods

### 2.1. Study Population and Data Collection

Data were collected from a cohort, the DFTJ cohort study which was launched in 2008 among retirees of Dongfeng Motor Corporation located in Shiyan City, Hubei, China, and has been described previously [14]. All study participants provided voluntary and written informed consent. The study protocol conformed to the ethical guidelines of the 1975 Declaration of Helsinki and was approved by the Tongji Medical College Ethics Committee, HUST, and Dongfeng General Hospital, Dongfeng Motor Corporation. The DFTJ cohort study is a prospective study in which 27,009 participants were followed from baseline assessments collected between 2008 and 2010. Individuals at baseline with any of the following were excluded from the study: presence of chronic hepatitis (*n* = 1,461); hepatic cirrhosis (*n* = 13), excessive alcohol consumption (more than 210 g/week for men or 140 g/week for women (*n* = 957)); or use of medications associated with NAFLD within the past two weeks such as valproate, amiodarone, or tamoxifen (*n* = 62). Persons with data missing at baseline for abdominal B-type ultrasound inspection (*n* = 892) were excluded. Additionally, 8,803 participants with measurements of liver fat at baseline were also excluded. Among 14,821 participants who finished the first follow-up in 2013, subjects with the presence of hepatitis B surface antigen (HBsAg, *n* = 3,013), cirrhosis (*n* = 2), use of any of the medications mentioned above (*n* = 3) or missing data on abdominal B-type ultrasound inspection (*n* = 63) were further excluded. Then, persons without data of SUA, age, or body mass index (BMI) or who were using SUA-lowering drugs were also removed from our analysis (*n* = 899). After exclusion, 8,429 participants were eligible for this analysis, and of these 2,007 subjects had developed incident NAFLD by the time of the follow-up examination.

Baseline information was collected from structured questionnaires via a face-to-face interview. Data on sociodemographic factors (age, sex, education, and marriage) and lifestyle were available from the questionnaires. A physical examination was also conducted at the same time to obtain baseline information on standing height, body weight, and waist circumference, which were measured with light indoor clothing and without shoes. The blood pressure was measured via a mercury sphygmomanometer in the morning. BMI was measured as weight (kilograms)/standing height (squared meters). After overnight fasting, fifteen milliliters of blood was obtained from participants and divided in three tubes (two ethylenediamine tetraacetic acid anticoagulation tubes for plasma and DNA and one coagulation tube for serum).

### 2.2. Determination of SUA and Variables

Measurement of SUA was based on colorimetric analyses conducted with an ARCHITECT ci8200 automatic analyzer (Abbott, USA) using Abbott Diagnostics reagents according to the manufacturer's recommendation. Serum total cholesterol (TC), triglycerides (TG), high-density lipoprotein cholesterol (HDL-C), low-density lipoprotein cholesterol (LDL-C)), alanine aminotransferase (ALT), aspartate aminotransferase (AST), gamma-glutamyl transferase (*γ*-GGT), urea nitrogen (Bun), and creatinine (Cre) were also determined by the same analyzer as SUA with corresponding reagent kits. Determination of fasting plasma glucose (FPG) and analysis of the complete blood constituents including platelet count (PLT) were conducted on an Aeroset automatic analyzer and CELL-DYN 3700 from Abbott Lab of USA, respectively. The AST to PLT ratio index (APRI) was computed as AST (upper limit of normal)/PLT (×10^9^/l) × 100. FIB-4 was calculated as age × AST (U/l)/(PLT (×10^9^/l)) × square root (ALT) (U/l) [15]. According to recommended cutoffs [16, 17], elevated levels were defined as follows: a serum ALT level > 30 U/l for men and >19 U/l for women, a serum *γ*-GGT level > 51 U/l for men and >33 U/l for women, APRI > 0.5 for both sexes, and FIB − 4 > 2.67 for both sexes. Those who currently or formerly smoked at least one cigarette per day for more than half a year were defined as smokers; otherwise, they were categorized as nonsmokers. A person who drank at least one time per day for more than half a year was categorized as a drinker. Correspondingly, drinking status was also classified into three groups: never drank, currently drinking, and quit. According to the participants' self-reported responses, physical activity was dichotomized as yes or no, and the classification of history of physician-diagnosed chronic diseases such as coronary heart diseases (CHDs), diabetes mellitus, and hypertension and the use of drugs (glucose lowering drugs) were also dichotomized. The presence of diabetes mellitus was defined based on the use of blood glucose-lowering medications or a fasting glucose ≥ 7.0 mmol/l or history of diabetes.

### 2.3. Assessment of NAFLD

NAFLD was defined as ultrasound diagnosed fatty liver using Aplio XG (TOSHIBA, Japan) performed by a unique independent specialist operator dedicated to abdominal ultrasound examinations. Hepatic steatosis was defined by the presence of at least two of three abnormal findings on abdominal ultrasonography: diffusely increased echogenicity (“bright”) liver with liver echogenicity greater than the kidney or spleen, vascular blurring, or deep attenuation [18]. Additionally, all individuals also met the following criteria: alcohol consumption < 30 g per day in men or 20 g per day in women; absence of HBV infection; without presence of chronic hepatitis and hepatic cirrhosis; or use of medications associated with NAFLD within the past two weeks such as valproate, amiodarone, or tamoxifen.

### 2.4. Genotyping

Several SNPs have been reported to be linked with SUA concentrations, and two SNPs (rs11722228 mapping to *SLC2A9* and rs2231142 mapping to *ABCG2*) were demonstrated to be significantly associated with elevated SUA levels in our previous study [19]. Therefore, these two SNPs were genotyped for 3,887 individuals using Affymetrix Genome-Wide Human SNP Array 6.0 chips in the iPLEX system (Sequenom, San Diego, USA) as previously described [20]. Both variants passed quality control criteria separately (call rates > 95% and MAF > 99.99). Genotypes of rs11722228 and rs2231142 were coded by applying an additive genetic model based on information from a GWSA [21]. None of the SNPs showed substantial deviation from the Hardy-Weinberg equilibrium among participants without NAFLD.

### 2.5. Statistical Analysis

The study fundamentally followed the checklist of the STROBE (Strengthening the Reporting of Observational Studies in Epidemiology) statement. Statistical analyses were performed using SAS software version 9.4. Categorical variables are expressed as percentages, and continuous variables are expressed as the mean ± SD unless otherwise specified. The simple and multivariable-adjusted odds ratios (ORs) of SUA levels for incident NAFLD were examined using logistic regression analysis. In the multivariable model, we adjusted for age; sex; BMI; physical activity; smoking and drinking status; presence of diabetes, CHD, and hypertension; and concentration of Cre, ALT, and FPG. The links between SUA and ALT, *γ*-GGT, APRI, and FIB-4 were conducted using covariance and logistic regression analysis for continuous and categorical variables, respectively. For genetic variants, we investigated deviation from the Hardy-Weinberg equilibrium using a Pearson *χ*^2^ test. Individual genotypes, common homozygotes, heterozygotes, and rare homozygotes were coded as 0, 1, and 2, respectively. The association between SNPs and SUA levels was examined using linear regression analysis adjusted for multivariable. The relation of SNPs with other NAFLD risk factors was investigated using linear regression and the Pearson *χ*^2^ test for continuous and categorical variables, respectively.

Finally, in Mendelian randomization, the causal link was determined by the difference between the observed effect sizes of SUA-related SNPs on NAFLD and the expected effect sizes. Logistic regression analysis was used to calculate ORs for the association between individual SNPs and incident NAFLD (observed effect size). The effect size of SNPs on SUA (*β*GB) and the effect size of SUA on NAFLD (*β*BD) were multiplied to compute the expected effect size (*β*E) of the individual SNP on incident NAFLD as the method previously reported [20]. The difference between the observed OR of individual SNPs on incident NAFLD and the expected OR was tested for statistical significance using an interaction test as described by Altman and Bland [22]. A two-tailed *P* value less than 0.05 was considered to be significant.

## 3. Results

### 3.1. Baseline Characteristics

Among 8,429 individuals, females were more likely to develop NAFLD than males. The concentration of SUA was significantly higher among participants with NAFLD than among those without NAFLD, and it varied substantially between females and males (Table S1). Increased SUA levels were markedly associated with increased age, BMI, FPG, TG, TC, LDL-C, ALT, AST, Bun, and Cre concentrations and mean blood pressure as well as decreased HDL-C levels (Table 1). The prevalence of diabetes, CHD, and hypertension was significantly higher than in persons in the top SUA quartile versus persons in the lowest quartile.

### 3.2. SUA Levels Associated with NAFLD

Compared to subjects in the lowest SUA level, subjects with the highest concentration showed an OR of 2.27 (95% CI: 1.96, 2.64, *P* < 0.001) for NAFLD incidence (Table 2). Adjusted for age, sex, BMI, smoking, drinking, and physical activity at baseline, subjects in quartile 4 showed an adjusted OR of 1.72 (95% CI: 1.46, 2.01, *P* < 0.001) for NAFLD incidence, compared to subjects in quartile 1. Moreover, the ORs were not further attenuated after additional adjustment, including controlling for the concentrations of Cre, FPG, and ALT and the prevalence of diabetes, hypertension, and CHD. When SUA was modeled as a continuous variable, the fully adjusted OR was 1.003 (95% CI: 1.002, 1.004, *P* < 0.001) per 1 *μ*mol/l or 1.18 (95% CI: 1.12, 1.24, *P* < 0.001) per 1 mg/dl. Furthermore, we observed a slightly more robust effect of SUA on incident NAFLD in women than in men. Notably, stratifying by BMI also revealed a pronounced association of SUA with incident NAFLD in normal-weight persons compared to overweight or obese persons.

### 3.3. Association between SUA and Suspected Progression of NAFLD

Serum ALT and *γ*-GGT, two markers of hepatic necroinflammation, as well as APRI and FIB-4 representing the estimated prevalence of fibrosis were used to investigate the association between SUA and suspected progression of NAFLD. Among patients with NAFLD, the mean ALT and *γ*-GGT levels gradually increased with increasing levels of SUA (Table 3). The prevalence of elevated serum ALT and GGT also increased with increasing SUA levels (Table 4). The corresponding ORs with multivariable adjustment of individuals in the highest quartile compared with those in lowest quartile were 1.57 (95% CI: 1.17, 2.11, *P* < 0.001) and 2.45 (95% CI: 1.67, 3.59, *P* < 0.001), respectively. A similar pattern was also observed in the association between SUA levels and the estimated development of fibrosis. In addition, the associations of SUA with ALT, *γ*-GGT, APRI, and FIB-4 were not substantially different among subgroups defined by sex and BMI (Table 4).

### 3.4. Mendelian Randomization Analysis

**Figure 1:**
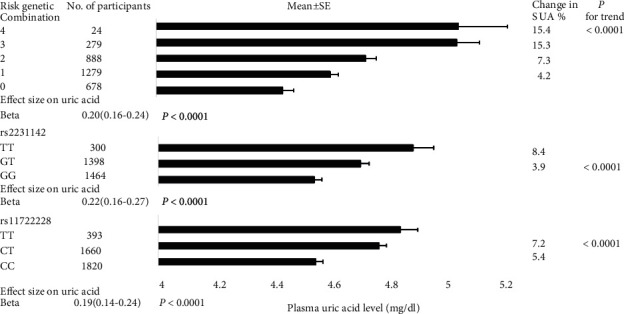
Levels of SUA as a function of SUA variant and variant combinations. Serum levels of SUA were measured, and rs1172228 at SLC2A9 and rs2231142 at ABCG2 were performed in 3800 participants. Beta values were determined after adjustment for variation in SUA levels due to age; sex; BMI; smoking status; drinking status; physical activity; ALT, FPG, and Cre concentrations; and prevalence of diabetes, CHD, and hypertension.

As shown in Figure 1, 7.2% and 8.4% of SUA concentration changes were accounted for individual variants rs11722228 and rs2231142, respectively. In a multivariable linear regression analysis, the association with SUA levels ranged from 0.19 mg/dl per risk allele for variant rs11722228 to 0.22 mg/dl per risk allele for variant rs2231142. Similarly, SUA levels continuously increased with each additional SUA-increasing allele in the genetic combination. Additionally, there was no evidence for a significant association between the variants and potential confounders of NAFLD (Table S2).

**Figure 2:**
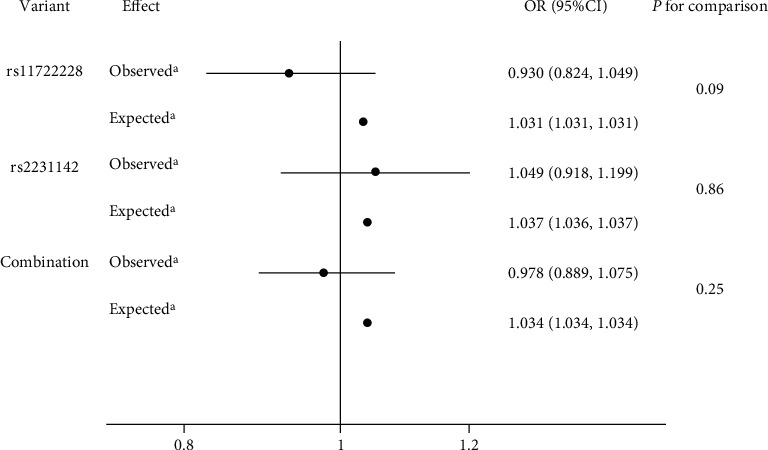
Comparison of observed and expected association of variant rs11722228 and/or rs2231142 with NAFLD. ORs with corresponding 95% CIs refer to a 59.5 *μ*mol/l (1 mg/dl) increase in SUA levels. Diamond indicates overall OR; horizontal lines indicate 95% CIs. ^a^Adjusted for age (continuous); sex (male and female); BMI (continuous) plus smoking (never/quit/currently smoking); drinking (never/quit/currently drinking); physical activity (yes/no); ALT, FPG, and Cre concentrations; and prevalence of diabetes, CHD, and hypertension (yes/no). *P* value for difference between expected and observed association with NAFLD.

The expected associations between variants rs11722228 and rs2231142 and between genetic combination and NAFLD were 1.031, 1.037, and 1.034, respectively. However, there were no significant associations between variations or genetic combinations and NAFLD. Furthermore, no significant difference between the expected associations and observed associations with NAFLD risk for individual SNPs or genetic combinations were observed (*P* range 0.09 to 0.86; Figure 2). Additionally, no evidence supported the significant association of the risk allele and NAFLD risk in either the both codominant or dominant model (Tables S3 and S4).

## 4. Discussion

First, our results revealed that increased SUA was stepwise and positively associated with the incidence of NAFLD. Furthermore, increased SUA levels were also associated with elevated levels of serum markers of hepatic necroinflammation and the estimated presence of liver fibrosis. However, the Mendelian randomization analysis supported no evidence for the causal association between SUA levels and the risk of NAFLD.

Similar to previous studies [4, 23], our findings support that SUA concentration is positively associated with NAFLD. Elevated UA contributes to the initiate of oxidative stress, insulin resistance, and metabolic syndrome and subsequently promotes NAFLD progression [24]. It even played a role in NAFLD induced by high fructose (e.g., in the form of sweetened beverages) [25]. In contrast, SUA-lowering medications such as allopurinol effectively reduced lipid accumulation in rats [26]. Additionally, a robust association of SUA levels with incident NAFLD risk among women compared to men was also observed. In the present study, most women were postmenopausal and decreased estrogen levels may partly contribute to sex differences [27]. Furthermore, we also observed an association varying substantially among subgroups defined by BMI. We suppose, as compared with overweight or obese persons, those with normal weight are more sensitive to SUA and subsequently susceptible to NAFLD. Future investigations are needed to clarify the mechanisms of the sex/BMI difference with regard to SUA and NAFLD risk.

Furthermore, our findings are partly consistent with and extend three earlier studies showing an independent association of hyperuricemia with ultrasonically diagnosed severe NAFLD (as opposed to mild or moderate NAFLD) [5], liver biopsy-proven NASH but not fibrosis [11], and cirrhosis development of unknown etiology [10]. To further explore the association of SUA with NAFLD development, we analyzed the association between normal SUA levels and the progression of NAFLD operationalized as hepatic necroinflammation and estimated the presence of liver fibrosis. As expected, a higher SUA level was associated with greater mean serum ALT and GGT levels and APRI and FIB-4 values, suggesting the influence of SUA on suspected progression of NAFLD. Thus, SUA may predict prognosis independently of other currently available predictors.

Even so, a crucial question is whether SUA plays a role in directly causing NAFLD incidence and progression or whether it is just a marker linked to NAFLD. Additionally, we cannot discount the possibility of residual confounding variables that were unlikely to be completely captured. The issues of the potential confounders and unclear cause-and-effect can be settled using a Mendelian randomization approach [13]. In contrast to thriving arguments concerning the causal effect of SUA on cardiovascular diseases and diabetes [28, 29], our study, for the first time, investigated the causality of SUA and NAFLD using instrumental variables. Both SNPs (s11722228 and rs2231142) are responsible for transporting and excreting urate [30]. Thus, both SNPs are consistently associated with SUA levels, which is in accordance with our study. Furthermore, our previous study also showed that s11722228 and rs2231142 explained 1.06% and 1.09% of the total variation in SUA levels, respectively [19]. However, our results do not support a causal role of SUA for NAFLD. Hence, these findings do not encourage the initiation of clinical trials or an expansion of serum-uric-acid-lowering interventions with the aim of preventing NAFLD. We suggest that SUA is a secondary phenomenon of an adverse metabolic phenotype, which was also suggested by Xu et al. [12] who first proposed the reversed causality of SUA and NAFLD risk.

Additionally, the present Mendelian randomization analyses could be underpowered. Although the present study had more than 99% power to detect ORs equals to 1.10 regarding the association of the individual SNPs with NAFLD risk, it had only 67.3% statistical power to detect the minor effects of the individual SNPs on the NAFLD risk. Therefore, our interpretation is cautious for NAFLD risk. Future studies will require large sample sizes to undertake analyses concerning the possible role of this genomic region and transport system, as well as the likely small effects of individual SUA-associated genetic variants on risk of NAFLD. Whether increased SUA is a cause or a marker of conditions that promote the incidence and progression of NAFLD is important, because pharmacological reduction of SUA levels is possible but will only be useful if SUA is a cause of these diseases.

There are potential limitations that bear mention. First, NAFLD was diagnosed by abdominal B-type ultrasound inspection, which is operator-dependent, thereby potentially underestimating the incidence rate of NAFLD due to invalid identification of progression of the NAFLD. Although liver biopsy is the gold standard, biopsy is difficult and unethical to perform in a health check-up. Nevertheless, ultrasonography is a widely accepted and cost-effective screening tool for NAFLD in population-based studies, with reasonable accuracy and sensitivity for detecting fatty liver [31]. Additionally, several markers weighing hepatic necroinflammation or fibrosis were simultaneously used to partly offset the limitations of ultrasonography insensitive to mild hepatic steatosis. Another limitation is that only two variants with small contributions to the total SUA concentration were genotyped in the present study. A recent genome-wide association study [32] identified nearly 30 gene variants for SUA, explaining approximately 7% of the total genetic variation. We believed that investigation of the effect of all SNPs related to SUA levels might improve statistical power. Finally, data on the presence of HBsAg infection were derived from 2013. Hence, the sample size of participants was perhaps overestimated, since the data needed to distinguish participants on the presence or absence of HBsAg antibodies at baseline were unavailable. Generally, this represents a small fraction of our total sample size and is unlikely to have materially altered our main results since the seroclearance of HBsAg was about 1.25% for 5-year follow-up according to a Taiwan study [33].

In summary, elevated SUA was associated with the increased development and suspected progression of NAFLD risk. The Mendelian randomization analysis lent no evidence for the causal roles of SUA in the development of NAFLD. Further studies examining the combined effect of more SNPs related to SUA with large sample sizes are warranted.

## Figures and Tables

**Table 1:** General characteristic of the study subjects according to SUA concentrations.

	Quartiles of SUA (mg/dl)				*P*
	Q1	Q2	Q3	Q4	
N	2085	2106	2101	2137	
Age	60.4 (7.6)	60.9 (7.4)	61.9 (7.8)	63.7 (8.0)	<0.001
Sex (female)	56.2	55.9	56.0	56.2	0.99
Education (≤6/7-9/10-12≥13)	29.1/38.8/23.0/9.1	28.7/36.8/24.8/9.7	28.0/34.9/24.9/12.3	30.7/34.2/22.8/12.4	<0.001
Waist circumference (cm)	78.2 (8.3)	79.7 (8.5)	80.4 (8.7)	81.9 (8.7)	<0.001
Body mass index (kg/m^2^)	22.6 (2.7)	23.1 (2.8)	23.5 (2.8)	24.0 (2.9)	<0.001
Fasting blood glucose (mmol/l)	5.86 (1.85)	5.70 (1.23)	5.76 (1.20)	5.83 (1.18)	<0.001
Triglyceride (mmol/l)	1.05 (0.55)	1.14 (0.60)	1.24 (0.84)	1.42 (0.85)	<0.001
Total cholesterol (mmol/)	5.00 (0.93)	5.07 (0.95)	5.13 (0.92)	5.20 (0.98)	<0.001
HDL (mmol/l)	1.51 (0.41)	1.48 (0.44)	1.45 (0.38)	1.39 (0.39)	<0.001
LDL (mmol/l)	2.93 (0.77)	2.99 (0.78)	3.04 (0.77)	3.11 (0.84)	<0.001
Alanine aminotransferase (U/l)	20.6 (24.0)	20.9 (12.1)	21.2 (10.8)	22.9 (16.0)	<0.001
Aspartate aminotransferase (U/l)	23.3 (14.3)	23.5 (10.0)	23.5 (7.7)	25.00 (11.5)	<0.001
Creatinine (*μ*mol/l)	75.5 (19.9)	77.4 (17.3)	80.2 (17.8)	87.4 (30.0)	<0.001
Urea nitrogen (*μ*mol/l)	5.00 (1.42)	5.08 (1.40)	5.21 (1.41)	5.56 (1.74)	<0.001
Systolic blood pressure (mmHg)	125.1 (17.6)	125.6 (18.3)	127.2 (18.2)	129.3 (18.4)	<0.001
Diastolic blood pressure (mmHg)	75.9 (10.4)	75.6 (10.7)	76.1 (10.6)	76.6 (10.8)	0.02
Smoking (current/quit/never)	18.7/9.7/71.5	18.0/9.6/72.5	16.1/12.0/71.9	14.7/11.9/73.4	0.001
Drinking (current/quit/never)	19.0/6.0/74.9	19.3/5.0/75.7	19.3/5.1/75.6	17.6/5.3/77.2	0.49
Physical activity (yes/no)	90.3/9.7	90.8/9.2	91.2/8.8	89.9/10.0	0.55
History of hypertension (%)	22.2	26.1	33.0	45.3	<0.001
History of coronary heart disease (%)	10.1	11.1	14.1	16.7	<0.001
Diabetes (%)	12.8	11.8	13.6	15.6	0.002

The quartiles of SUA concentration were computed sex, respectively. In male, the cutoff of SUA concentration is <4.47, ≥4.47, ≥5.23, and ≥6.10 mg/dl, respectively, and in female <3.53, ≥3.53, ≥4.15, and ≥4.84 mg/ml, respectively.

**Table 2:** Adjusted odds ratios and 95% confidence intervals for incident NAFLD by SUA quartiles.

	Quartiles of serum uric acid (mg/dl)				*P* for trend
	Q1	Q2	Q3	Q4	
Total					
Univariate model	Reference	1.44 (1.24, 1.69)	1.78 (1.52, 2.06)	2.27 (1.96, 2.64)	<0.001
Age and gender, adjusted	Reference	1.45 (1.24, 1.70)	1.79 (1.54, 2.08)	2.32 (2.00, 2.70)	<0.001
Multivariate model 1^a^	Reference	1.32 (1.12, 1.56)	1.44 (1.23, 1.69)	1.72 (1.46, 2.01)	<0.001
Multivariate model 2^b^	Reference	1.31 (1.11, 1.55)	1.43 (1.22, 1.69)	1.71 (1.45, 2.01)	<0.001
Male					
Univariate model	Reference	1.35 (1.05, 1.73)	1.45 (1.14, 1.86)	2.04 (1.61, 2.58)	<0.001
Age, adjusted	Reference	1.35 (1.05, 1.73)	1.46 (1.14, 1.86)	2.05 (1.61, 2.60)	<0.001
Multivariate model 1^a^	Reference	1.27 (0.98, 1.64)	1.22 (0.94, 1.57)	1.64 (1.28, 2.10)	<0.001
Multivariate model 2^b^	Reference	1.27 (0.98, 1.64)	1.22 (0.94, 1.58)	1.63 (1.26, 2.11)	<0.001
Female					
Univariate model	Reference	1.52 (1.24, 1.85)	2.00 (1.65, 2.43)	2.47 (2.04, 2.99)	<0.001
Age, adjusted	Reference	1.52 (1.25, 1.86)	2.02 (1.67, 2.46)	2.54 (2.09, 3.08)	<0.001
Multivariate model 1^a^	Reference	1.36 (1.10, 1.68)	1.60 (1.30, 1.97)	1.78 (1.44, 2.19)	<0.001
Multivariate model 2^b^	Reference	1.35 (1.09, 1.67)	1.59 (1.29, 1.96)	1.76 (1.42, 2.19)	<0.001
Normal-weight persons					
Univariate model	Reference	1.49 (1.17, 1.90)	1.76 (1.39, 2.24)	2.20 (1.74, 2.77)	<0.001
Age and gender, adjusted	Reference	1.49 (1.17, 1.91)	1.77 (1.39, 2.25)	2.24 (1.77, 2.83)	<0.001
Multivariate model 1^a^	Reference	1.49 (1.17, 1.91)	1.77 (1.39, 2.24)	2.24 (1.77, 2.83)	<0.001
Multivariate model 2^b^	Reference	1.50 (1.17, 1.93)	1.80 (1.41, 2.29)	2.23 (1.75, 2.85)	<0.001
Overweight/obese persons					
Univariate model	Reference	1.13 (0.92, 1.38)	1.17 (0.95, 1.43)	1.53 (1.26, 1.87)	<0.001
Age and gender, adjusted	Reference	1.13 (0.92, 1.39)	1.21 (0.98, 1.48)	1.63 (1.33, 2.00)	<0.001
Multivariate model 1^a^	Reference	1.14 (0.92, 1.40)	1.20 (0.98, 1.48)	1.63 (1.32, 2.00)	<0.001
Multivariate model 2^b^	Reference	1.10 (0.90, 1.36)	1.15 (0.93, 1.42)	1.56 (1.25, 1.93)	<0.001

The quartiles of SUA concentration were computed sex, respectively. In male, the cutoff of SUA concentration is <4.47, ≥4.47, ≥5.23, and ≥6.10 mg/dl, respectively, and in female <3.53, ≥3.53, ≥4.15, and ≥4.84 mg/ml, respectively. ^a^Adjusted for the age (continuous), sex (male and female), BMI (continuous) plus smoking (never smoking, quit smoking, and smoking), drinking (never drinking, quit drinking, and drinking), and physical activity (yes/no). In analysis of men and women, adjusted for age (continuous), BMI (continuous), smoking (never smoking, quit smoking, and smoking), drinking (never drinking, quit drinking, and drinking), and physical activity (yes/no). ^b^Adjusted for the same set of variables in model 1 plus prevalence (yes/no) of CHD, hypertension, diabetes, ALT concentration (continuous), Cre concentration (continuous), and FPG concentration (continuous).

**Table 3:** Association between SUA levels and elevated levels of serum ALT, GGT, APRI, or FIB4.

UA	Unadjusted mean difference in ALT	Adjusted^a^ mean difference in ALT	Unadjusted mean difference in GGT	Adjusted^a^ mean difference in GGT	Unadjusted mean difference in APRI	Adjusted^a^ mean difference in APRI	Unadjusted mean difference in FIB4	Adjusted^b^ mean difference in FIB4
Q1	0	0	0	0	0	0	0	0
Q2	2.26 (0.68, 3.84)	2.65 (1.05, 4.25)	5.15 (1.49, 8.82)	5.85 (2.09, 9.62)	0.02 (-0.00, 0.04)	0.02 (-0.00, 0.04)	0.02 (-0.09, 0.14)	-0.01 (-0.13, 0.11)
Q3	2.05 (0.48, 3.62)	2.63 (1.02, 4.24)	8.29 (4.64, 11.95)	8.75 (4.95, 12.55)	0.03 (0.01, 0.06)	0.04 (0.01, 0.06)	0.15 (0.04, 0.27)	0.13 (0.01, 0.25)
Q4	3.74 (2.16, 5.32)	4.53 (2.89, 6.18)	11.49 (7.82, 15.16)	12.48 (8.61, 16.35)	0.05 (0.03, 0.07)	0.05 (0.03, 0.07)	0.15 (0.04, 0.27)	0.13 (0.01, 0.25)

The quartiles of SUA concentration were computed sex, respectively. In male, the cutoff of SUA concentration is <5.26, ≥5.26, ≥6.08, and ≥7.16, respectively, and in female <4.27, ≥4.27, ≥4.99, and ≥5.76, respectively. ^a^Adjusted for sex (male and female), BMI (continuous) plus smoking (never smoking, quit smoking, and smoking), drinking (never drinking, quit drinking, and drinking), physical activity (yes/no), Cre concentration (continuous), FPG concentration (continuous), prevalence of hypertension, CHD and diabetes (yes/no). ^b^Adjusted for the age (continuous) plus variables in model ^b^.

**Table 4:** Association between SUA and elevated levels of serum ALT, GGT, APRI, or FIB 4 among NAFLD.

Quartiles of SUA	Elevated ALT (%)	Adjusted^a^ OR of elevated^c^ ALT	Elevated GGT (%)	Adjusted^a^ OR of elevated^c^ GGT	Elevated APRI (%)	Adjusted^a^ OR of elevated^c^ APRI	Elevated FIB 4 (%)	Adjusted^b^ OR of elevated^c^ FIB 4
Q1	28.37	Reference	10.26	Reference	6.04	Reference	11.07	Reference
Q2	30.10	1.08 (0.81, 1.45)	13.86	1.46 (0.99, 2.17)	7.72	1.43 (0.86, 2.37)	10.89	1.04 (0.69, 1.58)
Q3	31.00	1.17 (0.87, 1.57)	15.80	1.74 (1.18, 2.57)	9.20	1.71 (1.05, 2.81)	13.00	1.31 (0.87, 1.95)
Q4	35.64	1.57 (1.17, 2.11)	20.40	2.45 (1.67, 3.59)	14.65	2.92 (1.83, 4.67)	16.24	1.62 (1.09, 2.41)
Odds ratio per unit (mg/dl) increase in SUA								
All persons	N/A	1.14 (1.05, 1.24)	N/A	1.27 (1.15, 1.40)	N/A	1.32 (1.18, 1.48)	N/A	1.17 (1.05, 1.29)
Male	N/A	1.19 (1.03, 1.39)	N/A	1.26 (1.06, 1.50)	N/A	1.19 (1.01, 1.40)	N/A	1.12 (0.98, 1.29)
Female	N/A	1.13 (1.02, 1.25)	N/A	1.31 (1.15, 1.48)	N/A	1.47 (1.25, 1.74)	N/A	1.23 (1.05, 1.44)
Normal BMI	N/A	1.17 (1.02, 1.36)	N/A	1.33 (1.14, 1.57)	N/A	1.42 (1.16, 1.73)	N/A	1.22 (0.98, 1.51)
Over BMI	N/A	1.15 (1.06, 1.25)	N/A	1.28 (1.15, 1.40)	N/A	1.33 (1.18, 1.49)	N/A	1.16 (1.03, 1.30)

The quartiles of SUA concentration were computed sex, respectively. In male, the cutoff of SUA concentration is <5.26, ≥5.26, ≥6.08, and ≥7.16, respectively, and in female <4.29, ≥4.29, ≥5, and ≥5.78, respectively. Abbreviation: N/A: not applicable. ^a^Adjusted for the age (continuous), sex (male and female), BMI (continuous) plus smoking (never smoking, quit smoking, and smoking), drinking (never drinking, quit drinking, and drinking), physical activity (yes/no), Cre concentration (continuous), FPG concentration (continuous), and prevalence of hypertension, CHD, and diabetes (yes/no). ^b^Adjusted for the same multivariable of model ^a^ without age. ^c^An elevated ALT was a level > 30 U/l for male and >19 U/l for female; an elevated GGT level was a level > 51 U/l for male and >33 U/l for female; an elevated APRI level was >0.5 for both male and female, and elevated FIB-4 level was >2.67 for both male and female.

## Data Availability

The data, analytical methods, and study materials will be made available to other researchers for purposes of replicating the procedure and are available by contacting the corresponding authors.

## Abbreviations

NAFLD: Nonalcoholic fatty liver disease
HCC: Hepatocellular carcinoma
SUA: Serum uric acid
ALT: Alanine aminotransferase
*γ*-GGT: Gamma-glutamyl transferase
DFTJ: Dongfeng-Tongji
DMC: Dongfeng Motor Corporation
BMI: Body mass index
TC: Total cholesterol
TG: Triglycerides
HDL-C: High-density lipoprotein cholesterol
LDL-C: Low-density lipoprotein cholesterol
AST: Aspartate aminotransferase
Bun: Urea nitrogen
Cre: Creatinine
FPG: Fasting plasma glucose
PLT: Platelet count
CHD: Coronary heart diseases
OR: Odds ratio
95% CI: 95% confidence interval.

## Additional Points

*Highlights*. (1) Elevated serum uric acid was positively associated with NAFLD incidence, especially in female and normal-weight individuals. (2) A high concentration of serum uric acid increased the severity risk of newly developed NAFLD. (3) The Mendelian randomization analysis provided no evidence of a causal link between serum uric acid and NAFLD. (4) Our results suggest that high uric acid is a marker linked to NAFLD but does not play a role in directly causing NAFLD.

## Supplementary Materials

Supplementary Materials Table S1: the comparison of baseline parameters between subjects who developed or did not develop NAFLD. Table S2: the associations of variants with potential confounders. Table S3: the associations of uric acid-associated SNPs and variant combinations with NAFLD risk. Table S4: the associations of uric acid-associated SNPs and NAFLD risk in dominant model.
