# Supplementary material for: No Evidence for a Causal Link between Serum Uric Acid and Nonalcoholic Fatty Liver Disease from the Dongfeng-Tongji Cohort Study
Source: Oxid Med Cell Longev. 2022 Mar 15;2022:6687626. doi: 10.1155/2022/6687626 (PMC8941576; doi:10.1155/2022/6687626)
Supplement: Supplementary Materials — Table S1: the comparison of baseline parameters between subjects who developed or did not develop NAFLD. Table S2: the associations of variants with potential confounders. Table S3: the associations of uric acid-associated SNPs and variant combinations with NAFLD risk. Table S4: the associations of uric acid-associated SNPs and NAFLD risk in dominant model. [file 6687626.f1.zip › Table S1.docx]

**Table S1**. Comparison of baseline parameters between subjects developed or not developed NAFLD (mean ± SD, unless otherwise stated)

|  | All participants | NAFLD | | *P* |
| --- | --- | --- | --- | --- |
|  |  | Developed | No developed |  |
| N | 8429 | 2007 | 6422 |  |
| Age | 61.7(7.8) | 61.4 (7.8) | 61.9 (7.8) | 0.01 |
| Sex (female) | 56.1 | 64.1 | 53.6 | <0.001 |
| Education (≤6 /7-9/10-12≥13) | 29.1/36.2/23.9/10.8 | 31.2/36.35/22.1/10.2 | 28.4/36.0/24.4/11.1 | 0.03 |
| Waist circumference (cm) | 80.0 (8.7) | 83.5 (8.2) | 79.0 (8.5) | <0.001 |
| Body mass index (kg/m^2^) | 23.3 (2.8) | 24.9 (2.7) | 22.8 (2.7) | <0.001 |
| Uric acid (mg/dl) | 4.74 (1.29) | 4.92 (1.30) | 4.69 (1.29) | <0.001 |
| Fasting blood glucose (mmol/l) | 5.78 (1.39) | 5.91 (1.35) | 5.75 (1.40) | <0.001 |
| Triglyceride (mmol/l) | 1.22 (0.74) | 1.43 (0.95) | 1.15 (0.64) | <0.001 |
| Total cholesterol (mmol/l) | 5.10 (0.95) | 5.19 (0.96) | 5.07 (0.94) | <0.001 |
| HDL (mmol/l) | 1.46 (0.41) | 1.40 (0.36) | 1.48 (0.42) | <0.001 |
| LDL (mmol/l) | 3.01 (0.80) | 3.08 (0.82) | 3.00 (0.79) | <0.001 |
| Alanine aminotransferase (U/L) | 21.4 (16.5) | 22.7 (13.8) | 21.0 (17.3) | <0.001 |
| Aspartate aminotransferase (U/L) | 23.9 (11.1) | 23.3 (8.2) | 24.0 (11.9) | 0.002 |
| Creatinine (μmol/l) | 80.1 (22.4) | 79.1 (20.4) | 80.4 (22.9) | 0.01 |
| Urea nitrogen (μmol/l) | 5.22 (1.52) | 5.21 (1.53) | 5.22 (1.51) | 0.86 |
| Systolic blood pressure (mmHg) | 126.8 (18.2) | 128.3 (18.1) | 126.4 (18.2) | <0.001 |
| Diastolic blood pressure (mmHg) | 76.1 (10.6) | 76.9 (10.6) | 75.8 (10.6) | <0.001 |
| Smoking (current/quit/never) | 16.9/10.8/72.3 | 14.3/9.3/76.3 | 17.6/11.3/71.1 | <0.001 |
| Drinking (current/quit/never) | 18.8/5.4/75.8 | 17.45/4.7/78.7 | 19.2/5.5/75.3 | 0.07 |
| Physical activity (yes/no) | 90.5/9.5 | 89.5/10.5 | 90.9/9.1 | 0.07 |
| History of hypertension (%) | 31.7 | 39.1 | 29.4 | <0.001 |
| History of coronary heart disease (%) | 13.0 | 15.7 | 12.2 | <0.001 |
| Diabetes (%) | 13.5 | 16.8 | 12.4 | <0.001 |
