# Supplementary material for: No Evidence for a Causal Link between Serum Uric Acid and Nonalcoholic Fatty Liver Disease from the Dongfeng-Tongji Cohort Study
Source: Oxid Med Cell Longev. 2022 Mar 15;2022:6687626. doi: 10.1155/2022/6687626 (PMC8941576; doi:10.1155/2022/6687626)
Supplement: Supplementary Materials — Table S1: the comparison of baseline parameters between subjects who developed or did not develop NAFLD. Table S2: the associations of variants with potential confounders. Table S3: the associations of uric acid-associated SNPs and variant combinations with NAFLD risk. Table S4: the associations of uric acid-associated SNPs and NAFLD risk in dominant model. [file 6687626.f1.zip › Table S2.docx]

**Table S2.** Associations of variants with potential confounders.

|  | rs11722228 | *P* | rs2231142 | *P* |
| --- | --- | --- | --- | --- |
|  | effect size |  | effect size |  |
| Body mass index | 0.05(-0.08, 0.19) | 0.4319 | -0.06(-0.22, 0.09) | 0.4054 |
| waist circumference | -0.14(-0.44, 0.16) | 0.3693 | 0.32(-0.02, 0.65) | 0.66 |
| HDL | 0.01(-0.01, 0.03) | 0.1353 | -0.01(-0.03, 0.01) | 0.3108 |
| LDL | -0.01(-0.05, 0.03) | 0.9275 | -0.02(-0.07, 0.02) | 0.2960 |
| gender | 0.91(0.77, 1.07) | 0.2320 | 1.3(0.94, 1.37) | 0.1963 |
| drinking | 1.07(0.94, 1.22) | 0.2845 | 0.92(0.79, 1.07) | 0.2772 |
| smoking | 1.10(0.96, 1.25) | 0.1600 | 0.92(0.78, 1.07) | 0.2648 |
| Physical activity | 1.04(0.89, 1.21) | 0.6484 | 1.03(0.86, 1.23) | 0.7633 |
| Total cholesterol | -0.01 (-0.06, 0.03) | 0.9651 | -0.04(-0.09, 0.01) | 0.1025 |
| Triglyceride | -0.01(-0.03, 0.03) | 0.9933 | 0.01(-0.02, 0.05) | 0.4455 |
| Alanine aminotransferase | 1.12(0.16, 2.08) | 0.0218 | -0.95(-2.10, 0.19) | 0.1023 |
| Aspartate aminotransferase | -0.29(-0.68, 0.09) | 0.1369 | -0.13(-0.58, 0.31) | 0.5596 |
| Urea nitrogen | 0.07(0.01, 0.14) | 0.0321 | -0.02(-0.10, 0.05) | 0.5117 |
| Systolic blood pressure | -0.51(-1.30, 0.28) | 0.2255 | -0.07(-0.96, 0.82) | 0.8750 |
| Diastolic blood pressure | 0.03(-0.45, 0.52) | 0.8867 | 0.25(-0.29, 0.79) | 0.3599 |
| Creatinine | 0.77(-0.03, 1.56) | 0.0256 | 0.07(-0.83, 0.96) | 0.8872 |
| Fasting blood glucose | -0.03(-0.08, 0.02) | 0.1997 | 0.02(-0.03, 0.07) | 0.4687 |

Data are presented effect size: beta or OR (corresponding 95% CIs).
