# Supplementary material for: No Evidence for a Causal Link between Serum Uric Acid and Nonalcoholic Fatty Liver Disease from the Dongfeng-Tongji Cohort Study
Source: Oxid Med Cell Longev. 2022 Mar 15;2022:6687626. doi: 10.1155/2022/6687626 (PMC8941576; doi:10.1155/2022/6687626)
Supplement: Supplementary Materials — Table S1: the comparison of baseline parameters between subjects who developed or did not develop NAFLD. Table S2: the associations of variants with potential confounders. Table S3: the associations of uric acid-associated SNPs and variant combinations with NAFLD risk. Table S4: the associations of uric acid-associated SNPs and NAFLD risk in dominant model. [file 6687626.f1.zip › Table S3.docx]

**Table S3.** Associations between the uric acid associated SNPs and variants combination with NAFLD risk

|  | rs11722228 | | | rs2231142 | | | Variants combination | | | | |
| --- | --- | --- | --- | --- | --- | --- | --- | --- | --- | --- | --- |
|  | CC | CT | TT | GG | GT | TT | 0 | 1 | 2 | 3 | 4 |
| Incident NAFLD  N (%) | 432 (46.4) | 410 (44.0) | 81 (8.6) | 345 (45.0) | 352 (45.9) | 70 (9.1) | 155 (20.3) | 322 (42.2) | 224 (29.4) | 58 (7.6) | 4 (0.5) |
| without NAFLD  N (%) | 1388 (47.1) | 1250(42.4) | 312 (10.5) | 1119 (46.7) | 1046 (43.7) | 230 (9.6) | 523 (21.9) | 957 (40.1) | 664 (27.8) | 221 (9.3) | 20 (0.8) |
|  |  |  |  |  |  |  |  |  |  |  |  |
| Unadjusted OR (95% CI) | reference | 1.05  (0.90, 1.23) | 0.83  (0.64, 1.09) | reference | 1.09  (0.92, 1.30) | 0.99  (0.74, 1.32) | reference | 1.14  (0.91, 1.41) | 1.14  (0.90, 1.44) | 0.89  (0.63, 1.24) | 0.68  (0.23, 2.01) |
| *P* |  | 0.51 | 0.18 |  | 0.31 | 0.93 |  | 0.26 | 0.28 | 0.48 | 0.48 |
| Adjusted^a^ OR (95% CI) | reference | 0.99  (0.84, 1.17) | 0.80  (0.60, 1.07) | reference | 1.13  (0.94, 1.35) | 1.01  (0.74, 1.38) | reference | 1.14  (0.90, 1.44) | 1.12  (0.87, 1.44) | 0.90  (0.63, 1.30) | 0.51  (0.16, 1.62) |
| *P* |  | 0.94 | 0.13 |  | 0.20 | 0.94 |  | 0.27 | 0.38 | 0.58 | 0.25 |
| Adjusted^b^ OR (95% CI) | reference | 0.99  (0.84, 1.17) | 0.80  (0.60, 1.07) | reference | 1.13  (0.94, 1.35) | 1.01  (0.74, 1.38) | reference | 1.15  (0.91, 1.45) | 1.12  (0.87, 1.44) | 0.90  (0.63, 1.30) | 0.52  (0.30,1.64) |
| *P* |  | 0.88 | 0.13 |  | 0.21 | 0.96 |  | 0.26 | 0.39 | 0.58 | 0.26 |

a Adjusted for the age (continuous), sex (male, female), BMI (continuous) plus smoking (never smoking, quit smoking, currently smoking), drinking (never drinking, quit drinking, currently drinking), physical activity (yes/no).

b Adjusted for the same set of variables in model 1 plus Cre concentration (continuous), ALT concentration (continuous), glucose concentration (continuous), prevalence of CHD, hypertension and diabetes (yes/no).
