# Supplementary material for: No Evidence for a Causal Link between Serum Uric Acid and Nonalcoholic Fatty Liver Disease from the Dongfeng-Tongji Cohort Study
Source: Oxid Med Cell Longev. 2022 Mar 15;2022:6687626. doi: 10.1155/2022/6687626 (PMC8941576; doi:10.1155/2022/6687626)
Supplement: Supplementary Materials — Table S1: the comparison of baseline parameters between subjects who developed or did not develop NAFLD. Table S2: the associations of variants with potential confounders. Table S3: the associations of uric acid-associated SNPs and variant combinations with NAFLD risk. Table S4: the associations of uric acid-associated SNPs and NAFLD risk in dominant model. [file 6687626.f1.zip › Table S4.docx]

**Table S4.** Associations between the uric acid associated SNPs with NAFLD risk in dominant model

| variants | genotypes | Unadjusted OR (95% CI) | *P* | Adjusted^a^ OR (95% CI) | *P* | Adjusted^b^ OR (95% CI) | *P* |
| --- | --- | --- | --- | --- | --- | --- | --- |
| rs11722228 | CC vs (CT+TT) | 1.01 (0.87, 1.17) | 0.90 | 0.96(0.82, 1.12) | 0.57 | 0.95 (0.81, 1.11) | 0.53 |
| rs2231142 | CC vs (CT+TT) | 1.07 (0.91, 1.26) | 0.40 | 1.11(0.93, 1.32) | 0.26 | 1.10 (0.93, 1.32) | 0.27 |
| combination | (CC+GG) vs (CT+TT+GT+TT) | 1.10 (0.90, 1.35) | 0.35 | 1.10 (0.88, 1.36) | 0.40 | 1.10 (0.89, 1.36) | 0.40 |

a Adjusted for the age (continuous), sex (male, female), BMI (continuous) plus smoking (never smoking, quit smoking, currently smoking), drinking (never drinking, quit drinking, currently drinking), physical activity (yes/no).

b Adjusted for the same set of variables in model 1 plus Cre concentration (continuous), glucose concentration (continuous), ALT concentration (continuous), prevalence of hypertension, CHD and diabetes (yes/no).
